# Analysis of the morphology of retinal vascular cells in zebrafish (*Danio rerio*)

**DOI:** 10.3389/fcell.2023.1267232

**Published:** 2023-10-02

**Authors:** Chiara Simone Middel, Nadine Dietrich, Hans-Peter Hammes, Jens Kroll

**Affiliations:** ^1^ Department of Vascular Biology and Tumor Angiogenesis, European Center for Angioscience, Medical Faculty Mannheim, Heidelberg University, Mannheim, Germany; ^2^ Fifth Medical Department and European Center for Angioscience, Medical Faculty Mannheim, Heidelberg University, Mannheim, Germany

**Keywords:** zebrafish retina, endothelial cells, pericytes, vascular mural cells, diabetic retinopathy, quantitative retinal morphometry

## Abstract

**Background:** Zebrafish (*Danio rerio*) have been established in recent years as a model organism to study Diabetic Retinopathy (DR). Loss of endothelial cells and pericytes is an early hallmark sign of developing DR in the mammalian retina. However, morphology, numbers, ratios, and distributions of different vascular cells in the retinal compartment in zebrafish have not yet been analyzed and compared with the mammalian retina.

**Methods:** The retinal trypsin digest protocol was established on the zebrafish retina. Cell types were identified using the *Tg(nflk:EGFP)-*reporter line. Cells were quantified using quantitative morphometry.

**Results:** Vascular cells in the zebrafish retina have distinct morphologies and locations. Nuclei of vascular mural cells appear as long and flat nuclei located near the vessel wall. Round nuclei within the vessel walls can be identified as endothelial cells. The vessel diameter decreases from central to peripheral parts of the retina. Additionally, the numbers of vascular cells decrease from central to peripheral parts of the retina.

**Discussion:** The retinal trypsin digest protocol, which can be applied to the zebrafish retina, provides novel insights into the zebrafish retinal vascular architecture. Quantification of the different cell types shows that, in comparison to the mammalian retina, zebrafish have higher numbers of mural cells and an increased mural cell to endothelial cell ratio. This protocol enables to quantify mural cell and endothelial cell numbers, is easily adaptable to different transgenic and mutant zebrafish lines and will enable investigators to compare novel models on a single cell level.

## 1 Introduction

Multiple ocular pathologies are associated with changes in the retinal vasculature. Diabetic retinopathy is one of the leading causes of vision impairment or blindness globally (Bourne et al., 2013) and one of the main microvascular complications associated with both type 1 and type 2 diabetes mellitus (Antonetti et al., 2021). The main pathobiology behind the development of diabetic retinopathy has been described as the “unifying hypothesis” (Brownlee, 2005). Cell types which are unable to downregulate the transport of glucose into the cell during hyperglycemic conditions, such as endothelial cells, are damaged by hyperglycemia induced formation of reactive oxygen species leading to oxidative stress and inflammation (Brownlee, 2005; Madsen-Bouterse and Kowluru, 2008) and the breakdown of the blood-retinal barrier (Antonetti et al., 2021).

Improved understanding of the cellular mechanisms of diabetic retinopathy has shown that disruption of the neurovascular unit is critically involved in the pathogenesis (Gardner and Davila, 2017). The term “neurovascular unit” refers to the intricate interactions between vascular cells, which interact to regulate the retinal blood flow in accordance with metabolic demands (Kur et al., 2012). Cell types include endothelial and mural cells, neural cells, which in the retina include ganglion, amacrine, horizontal and bipolar cells, as well as macroglia, i.e., Müller glia cells, and microglia (Metea and Newman, 2007). Especially interactions between endothelial cells and vascular mural cells are disrupted early in the pathogenesis of diabetic retinopathy (Duh et al., 2017).

Early structural changes in diabetic retinopathy are the loss of pericytes and consecutively the loss of endothelial cells, leading to vasoregression and, when progressing, decreased blood flow in the retina (Hammes, et al., 2004). In the human retina, retinal ischemia is followed by an angiogenic response leading to the formation of new blood vessels and the clinical phenotype of proliferative diabetic retinopathy (Hammes, et al., 2011).

In recent years, zebrafish (*Danio rerio*) have come into focus as a convenient animal model to study diabetic complications (Heckler and Kroll, 2017). Diabetic microvascular complications are the main point of interest, as the use of the *Tg(fli1a:EGFP)* reporter line (Lawson and Weinstein, 2002) facilitates the visualization of vessels and the identification of changes in the vasculature. Multiple pathologies associated with diabetic retinopathy have been studied in the zebrafish retina (Middel, et al., 2021). In zebrafish, a diabetic phenotype can be achieved through inactivation of genes (Wiggenhauser and Kroll, 2019). Multiple zebrafish lines have been shown to exhibit increased angiogenesis in the retina due to hyperglycemic conditions, including the *pdx1* ^*−/−*^ zebrafish line, the *aldh2.1* ^−/−^ zebrafish line and the *glo1* ^*−/−*^ zebrafish line (Lodd et al., 2019; Ali, et al., 2020; Wiggenhauser, et al., 2020; Schmitner, et al., 2021; Wohlfart, et al., 2022).

It has already been shown that pericytes and endothelial cells exist in the zebrafish retina (Alvarez, et al., 2007; Caceres, et al., 2019; Ali, et al., 2020). Recently, the retinal trypsin digest has been performed on the zebrafish retina for the first time. Using cell type specific antibody staining, the existence and precise location of endothelial cells and vascular mural cells were shown in detail (Lee, et al., 2022). However, the standard method to quantitate vascular cells in the mammalian retina, quantitative retinal morphometry (Dietrich and Hammes, 2012), has not yet been adapted to the zebrafish retina.

In this study, after performing the retinal trypsin digest protocol on the zebrafish retina, we analyzed the morphology of the retinal vascular cells using only Mayer’s hemalum stain and performed quantitative morphometry on wildtype zebrafish.

## 2 Materials and equipment

### 2.1 Equipment

• Cell-F software (Olympus Opticals)
• Conical tubes (15 mL, 50 mL, Falcon)
• Cover slips (24 × 60 mm, Menzel-Gläser)
• Culture dishes (35 × 10 mm, 60 × 15 mm, Thermo Fisher Scientific)
• Dumont Tweezers No. 5 (neoLab)
• Feather disposable scalpel No. 10 (Feather)
• Leica TCS SP5 Confocal Microscope (Leica)
• Microscissors Vannas 2.5 mm (Fine Science Tools)
• Microscope slides (76 × 26 mm) (neoLab)
• Olympus BX51 Microscope (Olympus)
• Safe-lock tubes (0.5, 1.5 and 2 mL, Eppendorf)
• Syringes (10 mL, BD Plastik)
• XC10 camera (Olympus)

### 2.2 Chemicals

**Table:** 

E3 (“eggwater”)	3 g Red Sea Salt
	ad 10 L MilliQ^®^ water
1-phenyl-2-thiourea (PTU, 10x stock)	304 mg PTU
	ad 1 L MilliQ^®^ water
PTU working solution	100 mL PTU 10x stock
	Ad 900 mL MilliQ^®^ water
10x PBS	400 g NaCl
	10 g KCl
	57.5 g Na_2_HPO_4_
	10 g KH2PO_4_
	ad 5 L MilliQ^®^ water
1x PBS	1 L 10x PBS + 9 L MilliQ^®^ water
4% Formalin	100 mL 37% formalin
	100 mL 10x PBS
	fill up to 1 L with Aqua bidest
3% Trypsin	1.5 g trypsin (1:250, Sigma-Aldrich)
	50 mL 0.2M Tris-HCl, pH 7.45
Mayer’s hemalum solution	ready-made (Millipore).
DPX mounting medium	ready-made (Thermo Fisher Scientific)
≥99.8%/96%/80%/70% ethanol	ready-made (Carl Roth)
Xylene (isomeres)	ready-made (Carl Roth)

## 3 Methods

### 3.1 Zebrafish husbandry and zebrafish lines

Zebrafish embryos of the *Tg(nflk:EGFP)* (Blum, et al., 2008) line were raised in E3 medium (“egg water”) at 28.5°C with 0.003% 1-phenyl-2-thiourea (PTU) to suppress pigmentation. In the *Tg(nflk:EGFP)* transgenic line, GFP is expressed in nuclei of endothelial cells under the control of the *flk1/vegfr2* promotor, which is activated early during the development of endothelial cells (Jin, et al., 2005). Zebrafish were staged as described (Kimmel, et al., 1995) and were referred to as larvae starting at >72 h postfertilization (hpf). At 6 days postfertilization (dpf) the larvae were transferred to adult boxes and kept under a 13 h light/11 h dark cycle. At 90dpf, the zebrafish were referred to as adult. Adult zebrafish were fed daily with living shrimps (*Artemia Salina*) in the morning and fish flake food in the afternoon. For these experiments, zebrafish from the *Tg(nflk:EGFP)* line were used at >24 months post fertilization (mpf). The zebrafish were sacrificed through hypothermal shock in 2°C–4°C cold water (Wilson, et al., 2009).

### 3.2 Retinal digest preparation

The eyes were carefully extracted from the head at the optic nerve and fixed in 4% formalin at room temperature for 48 h. Retina dissection was performed as previously described by our laboratory (Wiggenhauser, et al., 2017). The preparation of the retinal trypsin digest was performed based on an established protocol (Dietrich and Hammes, 2012) with slight adjustments. After dissection, the retina was placed inside a culture dish with aqua bidest and incubated at 37°C overnight. After overnight incubation the retina was rinsed twice in changes of clean aqua bidest and finally transferred into a culture dish with 3% porcine trypsin in 0.2 mol/L Tris-HCl buffer and incubated again at 37°C for 1.5 h. After the incubation period, the retina was transferred onto a microscope slide with the retinal pigment epithelium (RPE) side up and the vasculature in direct contact with the microscope slide. The retinal cells were cleared from the vasculature by dropping aqua bidest with a syringe onto the retina and removing them from the slide using an aspirator. The procedure was monitored under a microscope to prevent accidental aspiration of the vasculature. After removal of the retinal cells, the digest preparations were left to air-dry for approximately half an hour.

### 3.3 Confocal microscopy

To identify endothelial cells in the retinal vasculature, confocal images of the air-dryed digest preparations before staining with Mayer’s hemalum solution (Millipore) were acquired using a confocal microscope (DM6000 B) with a scanner (TCS SP5 DS). Images of distinct areas were taken with a ×20 water immersion objective, 400 Hz bidirectional, 1,024 × 1,024 pixels and z-stack steps of 1.5 µm thickness.

### 3.4 Hemalum stain

To properly visualize the different cell types in the retina, nuclei staining with undiluted Mayer’s hemalum solution (Millipore) was performed. After air-drying the preparations, the slides were shortly placed in aqua bidest and then moved to fresh undiluted Mayer’s hemalum solution for 7 min. Afterwards, they were placed in lukewarm tap-water for 2 min and then moved shortly to 70% and then 80% ethanol before being put in 96% and then 99.8% ethanol for 5 min each. After the last change of ethanol, the slides were placed in two changes of xylene and kept there for 5 min each before the slides were covered with cover slips using DPX mounting medium (Thermo Fisher Scientific). Hemalum stained samples can be kept at room temperature for approximately 2 weeks without significant loss of quality.

### 3.5 Morphological quantification

To determine the numbers and morphologies of retinal vascular mural cells and endothelial cells, the retinal digest preparations were analyzed using the image analyzing system Cell-F (Olympus Opticals). To establish the number of endothelial cells and vascular mural cells in the retina of healthy adult zebrafish images of all areas of the retina were taken with an Olympus BX51 microscope (Olympus Opticals) under ×200 magnification. Images were taken in all density areas of the retina (high density, medium density, low density) and in central, intermediate, and peripheral parts of the retina (Figure 3). Nuclei were counted over 200 µm of the vessel and the vessel diameter was measured to allow for standardization to the capillary density (numbers of cells per mm^2^ of capillary area).

### 3.6 Statistics

Results are expressed as mean ± SD. One-way ANOVA followed by appropriate multiple comparison tests was used to analyze statistical significance between different groups. Statistical analyses were performed using GraphPad Prism software. A *p*-value of *p* < 0.05 was considered as significant.

### 3.7 Study approval

Experimental procedures on zebrafish were approved by Medical Faculty Mannheim (License no. I-21/18). All experiments were carried out in accordance with the approved guidelines.

## 4 Results

### 4.1 Adaptation of the retinal trypsin digest protocol to the zebrafish retina

The application of the retinal digest protocol on mouse retinas has provided very valuable information about pathological retinal cellular changes under diabetic conditions for decades (Hammes, et al., 2002; Pfister, et al., 2013; Hammes, 2018). Since not all aspects of DR can be analyzed in rodents, the study aimed to provide a detailed morphological analysis of the zebrafish retina using an adapted retinal digest protocol.

Some technical challenges arose during the adaptation of the retinal trypsin digest protocol to the zebrafish retina. The retinal vasculature in zebrafish is located on top of the inner limiting membrane (ILM) and the vessels are tightly connected to the ILM. While the ILM is removed during the digestion process in the mammalian retina, removal of the ILM in zebrafish led to removal of the vasculature. Therefore, this experimental step for the zebrafish retina digestion was skipped. Since the remaining zebrafish ILM consists mainly of collagen and showed a strong background color after hemalum stain, we also needed to adapt the quantification of the capillary area and number of cells per mm^2^, which is now provided manually.

**FIGURE 1:**
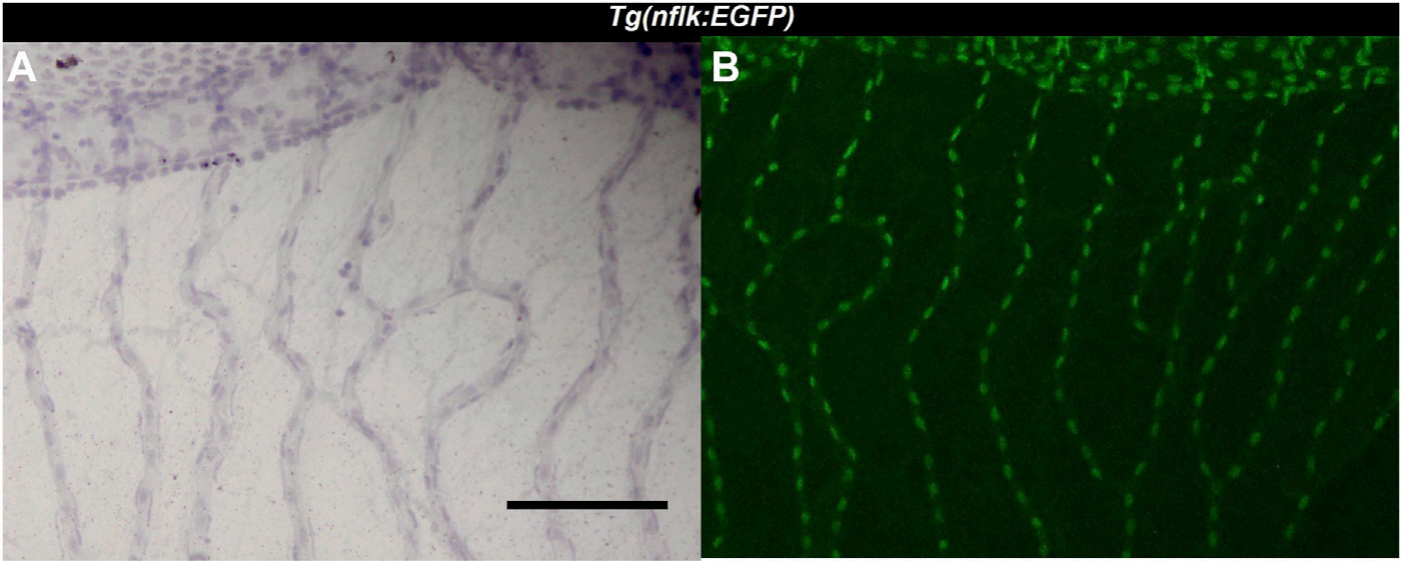
Identification of endothelial cells using the *Tg*(*nflk:EGFP*) zebrafish line. **(A)**: Image of the zebrafish retina at ×200 magnification after hemalum stain taken with a light microscope. Scale bar: 100 µm. **(B)**: Image of the same zebrafish retina taken with a confocal microscope directly after digestion.

The intermediate part of the zebrafish retina was the most reliably quantifiable part (Figure 1). In the central part, the high numbers of cells and the increased vessel diameter led to overlaps between the cells which could make it difficult to decisively identify the different cell types. During preparation and digestion, damage sometimes occurred in the peripheral part of the retina, which complicated analyzing the same number of images per retina. We found that the middle part of the retina was usually well preserved, and the cells were spaced out along the vessels, making it easier to identify and quantify the different cell types. The vessel diameter in the intermediate area of the zebrafish retina varied significantly. Therefore, to ensure comparability not only with the mammalian retina but also between experimental groups, it was necessary to quantify vascular cells in the zebrafish retina as cells per mm^2^.

### 4.2 Endothelial cells and mural cells showed distinct morphologies and locations in the zebrafish retina

**FIGURE 2:**
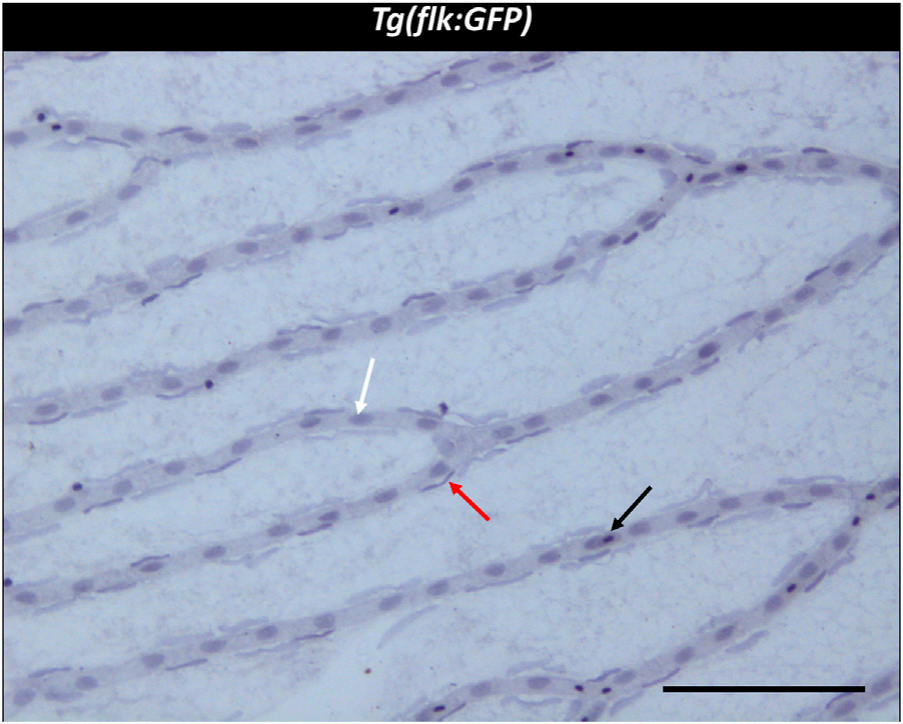
Trypsin digest of the zebrafish retina. ×200 magnification of a zebrafish retinal trypsin digest preparation. Black arrow: Erythrocyte. White arrow: Endothelial cell. Red arrow: vascular mural cell. Scale bar: 100 µm.

**FIGURE 3:**
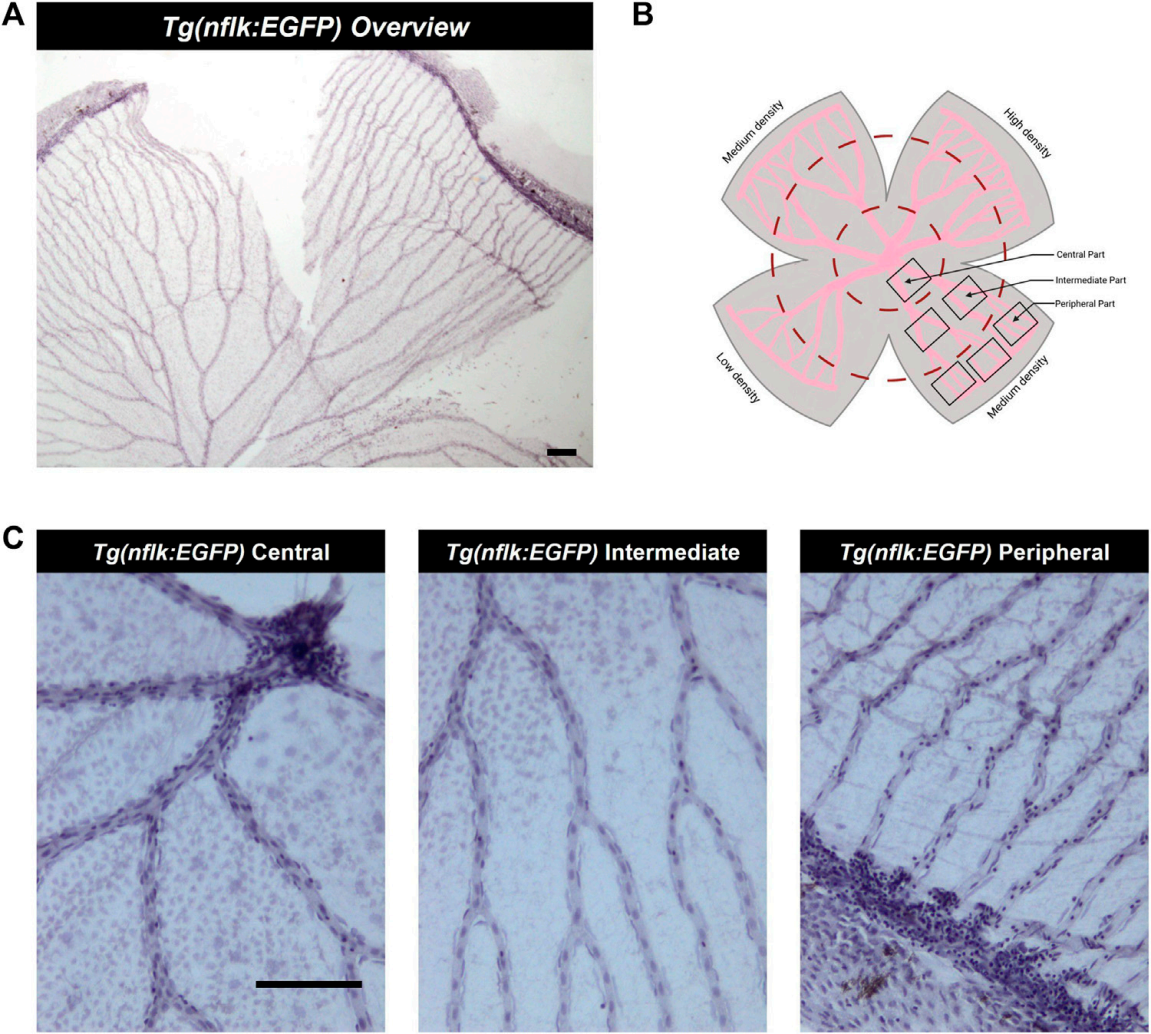
Analysis of the adult zebrafish retina according to density areas and distance to the entrance of the optic artery into the retina. **(A)** Overview image of a retinal trypsin digest preparation, ×40 magnification. Scale bar: 100 µm. **(B)** Schematic overview of the areas analysed. **(C)** Representative images of the different parts of the retina (central, intermediate, peripheral), ×200 magnification. Scale bar: 100 µm. This figure was in part created with BioRender.com.

Three different cell types were discernible in the vessels of the zebrafish retinal trypsin digest (Figure 2). The nuclei of one of the cell types we identified were located with some distance to the vessel wall. The nuclei were long, flat, and stained dark purple. Morphology and location resembled the cells that had been described as vascular mural cells (vMC) in the zebrafish retina before (Caceres, et al., 2019; Lee, t al. 2022). Small and round nuclei that were stained nearly black were located both between the vessel walls and around the vessels. We identified these cells as erythrocytes, based on their staining characteristics, their location, and the fact that zebrafish erythrocytes contain nuclei thus stained dark by a hemalum. Between the vessel walls were oval nuclei of a light purple color after staining with Mayer’s hemalum solution. To verify that these cells were endothelial cells, we used the transgenic *Tg(nflk:EGFP)* zebrafish line in which the nuclei of endothelial cells express endothelial green fluorescence protein (EGFP). Retinae of *Tg(nflk:EGFP)* zebrafish were processed using the digest protocol described above. Prior to staining, confocal images were taken, and matched with digest preparations after staining. Respective images were compared in specific locations allowing for the unequivocal identification of endothelial cells as the third cell type (Figure 3).

In conclusion, we were able to successfully identify all cell types in the zebrafish retinal digest preparation. We also showed that all cell types which play a role in the pathogenesis of human retinal vascular pathologies are present in the zebrafish retina and can be quantified.

### 4.3 Vessel diameter decreased from central to peripheral parts of the zebrafish retina

In the zebrafish retina, the optic artery splits into 5–7 main vessels at the entrance into the retina which divide twice on average before connecting in the periphery. They form the circumferential vein resulting in the inner optic circle (IOC). We defined the main vessels before they divide as vessels in the “central” part of the retina, the vessels between the first division point and the second division point as vessels in the “intermediate” part of the retina and the vessels which were connected to the IOC as vessels in the “peripheral” part of the retina. Furthermore, the zebrafish retina has areas with differing vascularity due to an uneven pattern of intervascular distance in the periphery of the retina. There is one high density, one low density and two medium density areas. Highest and lowest vascularity are located opposite each other (Figure 1).

To ensure comparability between zebrafish and mammalian retinal vascular cell numbers it was necessary to calculate the number of endothelial cells and vMCs per mm^2^. To establish the unit cells/mm^2^ we counted the cells over 200 µm of vessel length and measured the vessel diameter to calculate the area.

We found that the vessel diameter decreased from central to peripheral parts of the retina. In the central part of the retina, vessels had a diameter of on average 15.13 ± 1.96 µm, which decreased to 12.22 ± 1.61 µm in the intermediate area and to 9.99 ± 1.12 µm in the peripheral area. If compared to the mammalian retina, only the vessels in the periphery of the retina would be qualified as capillaries, while the vessels in the intermediate and central parts of the retina would be arterioles (Holm, et al., 2018).

**FIGURE 4:**
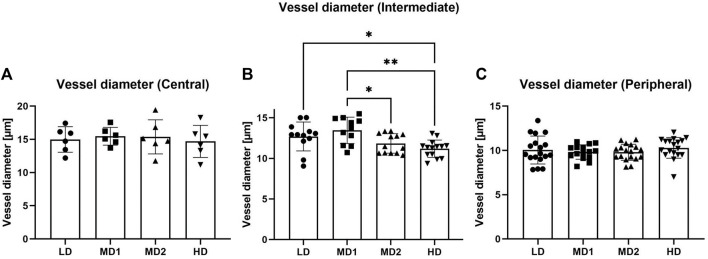
The vessel diameter varies between the different areas in the intermediate part of the retina. **(A–C)** Vessel diameter [µm] in the different parts of the retina according to density areas. Abbreviations: LD, low density area; MD1/2, medium density area 1/2; HD, high density area.

Furthermore, we found that the vessel diameters varied between the different density areas in the intermediate part of the retina while they remained comparable in the central and peripheral part of the retina (Figure 4).

In conclusion, we established a method to count the different cell types. We discovered that due to the variation in vessel diameter between different density groups in the intermediate part of the retina it is essential to quantify cells in the zebrafish retina as cell numbers per mm^2^ to ensure comparability.

### 4.4 Decline of endothelial cells and vMCs from the central to the peripheral part of the retina

To conclusively analyse the retinal vascular architecture, we quantified vascular cell numbers in all areas of the retina of adult zebrafish.

**FIGURE 5:**
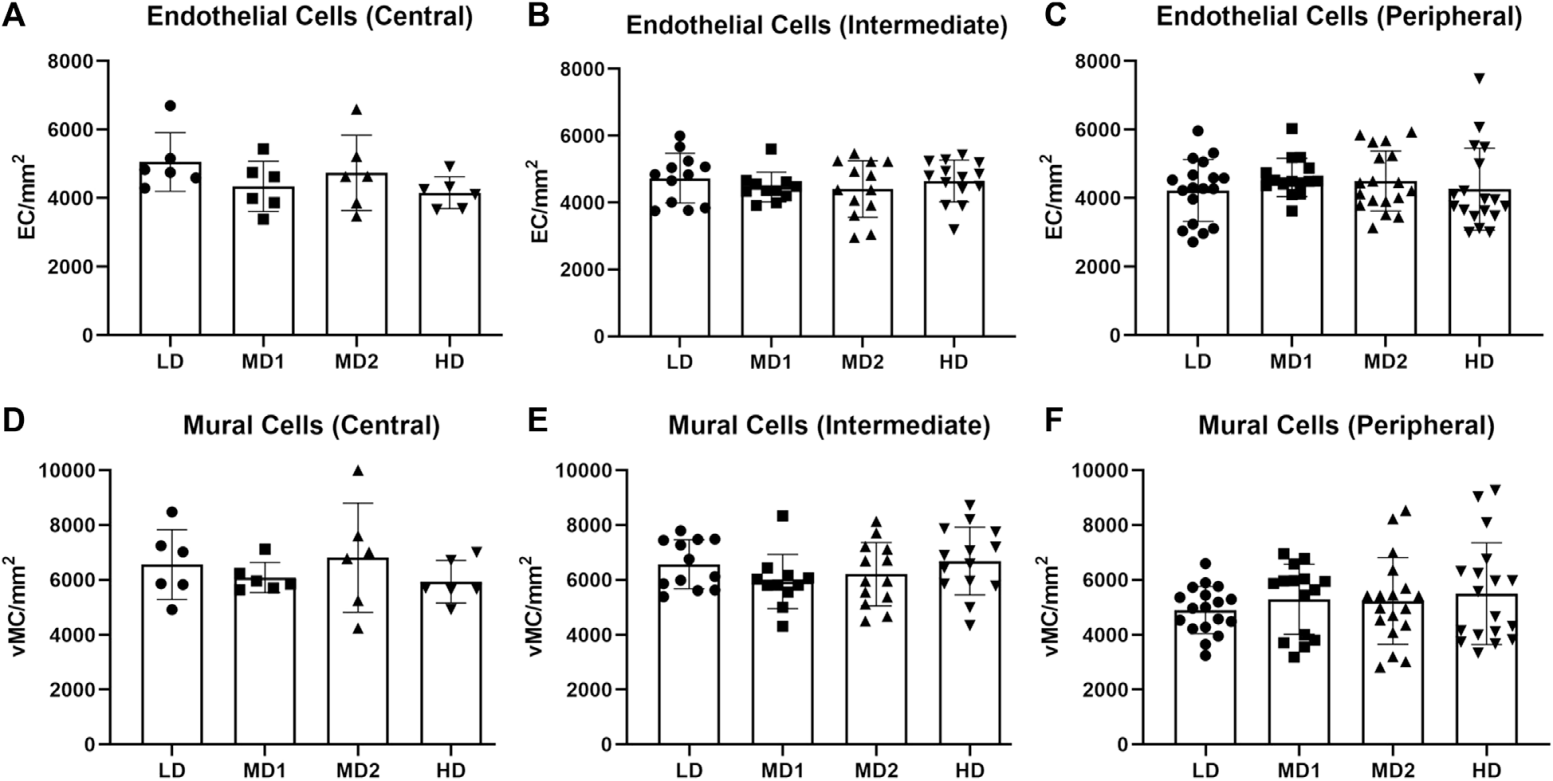
The number of endothelial cells and mural cells per mm^2^ does not vary between the different areas of the retina when calculated in the correlating parts. **(A–C)** Number of endothelial cells per mm^2^ in the central, intermediate and peripheral parts of the retina according to density area. **(D–F)** Number of mural cells per mm^2^ in the central, intermediate and peripheral parts of the retina according to density area. Abbreviations: EC, endothelial cell; vMC, vascular mural cell; LD, low density area; MD1/2, medium density area 1/2; HD, high density area. *n* = 6 zebrafish eyes. Central area: 1 image/density area/eye, intermediate area: 2 images/density area/eye, peripheral area: 3 images/density area/eye.

Per retina, we analysed four images taken in the central part, eight images taken in the intermediate part and twelve images taken in the peripheral part (Figure 1). The numbers of endothelial cells and vMCs per mm^2^ vascular area decreased significantly from central to peripheral parts of the retina (endothelial cells: 4561.56 ± 810.18/mm^2^ central vs. 4297.40 ± 932.50/mm^2^ peripheral; vMCs: 6174.49 ± 1333.33/mm^2^ central vs. 5078.31 ± 1420.27/mm^2^ peripheral). This variability in cell densities of the different parts of the retina requires careful pre-test definition of which areas are compared (Figure 5).

### 4.5 No difference in endothelial cell or vMC numbers regarding the retinal area

To account for differences between the density areas, we counted endothelial cells and vMCs in each area (Figure 5). We found that there was no significant difference in the number of endothelial cells per mm^2^ or the number of vMCs per mm^2^ vascular area between the different areas when analyzed in the same part (central, intermediate, or peripheral) of the retina (Table 1). We interpreted this observation as confirmation that retinal vascular cells in a healthy adult retina are evenly spread over the vasculature.

**TABLE 1:** Results of quantitative retinal morphometry in adult zebrafish. *n* = 6 zebrafish eyes.

Retina part	High density area	Medium density area 1	Medium density area 2	Low density area
Number of vessels (average ±standard deviation)				
Central	2.17 ± 0.37	2.33 ± 0.75	2.17 ± 0.37	2.50 ± 0.50
Intermediate	3.14 ± 0.64	3.09 ± 0.99	3.08 ± 0.47	3.00 ± 0.41
Peripheral	3.89 ± 0.74	4.13 ± 0.81	3.89 ± 0.57	4.06 ± 0.97
Capillary area [mm^2^]/image (average ±standard deviation)				
Central	0.0059 ± 0.0016	0.0070 ± 0.0025	0.0062 ± 0.0011	0.0070 ± 0.0023
Intermediate	0.0072 ± 0.0013	0.0080 ± 0.0022	0.0076 ± 0.0012	0.0077 ± 0.0010
Peripheral	0.0081 ± 0.0017	0.0087 ± 0.0015	0.0079 ± 0.0011	0.0081 ± 0.0022
Endothelial cells/mm^2^ (average ±standard deviation)				
Central	4411.10 ± 505.61	4360.66 ± 978.67	4834.92 ± 943.97	4639.56 ± 614.23
Intermediate	4454.95 ± 646.96	4308.56 ± 511.17	4243.97 ± 807.91	4890.32 ± 656.26
Peripheral	4164.32 ± 1174.37	4183.91 ± 389.01	4484.45 ± 882.24	4338.02 ± 992.29
Mural cells/mm^2^ (average ±standard deviation)				
Central	6160.09 ± 570.06	6009.16 ± 1254.03	6721.60 ± 1788.41	5807.12 ± 1246.30
Intermediate	6351.54 ± 1396.93	5212.06 ± 835.28	5928.89 ± 1318.19	6663.11 ± 999.65
Peripheral	5129.97 ± 1607.21	4526.25 ± 1177.76	5490.05 ± 1618.16	5074.94 ± 974.06

### 4.6 The number of mural cells per mm^2^ capillary area in zebrafish was higher than the number of mural cells per mm^2^ capillary area in mammals

**FIGURE 6:**
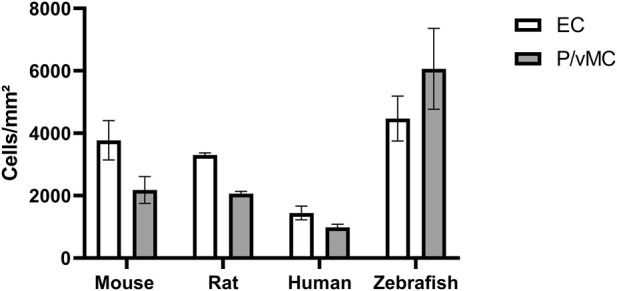
Zebrafish have higher absolute numbers of endothelial cells and vascular mural cells than mammals and a higher vascular mural cell to endothelial cell ratio. Abbreviations: EC, endothelial cells; P, pericytes (mouse/rat/human); vMC, vascular mural cells (zebrafish). The data from mouse, rat and human were originally described in (Hammes, 2018).

Pericyte loss as a hallmark sign of DR has been studied extensively in mammals. Control animals usually have a pericyte count of around 2,200 pericytes per mm^2^ capillary area in rodent models of diabetic retinopathy (Hammes, 2018). We found that in the intermediate part of the retina, zebrafish have on average 6065.75 ± 1293.80 mural cells per mm^2^ capillary area (Table 1). In the mammalian retina the mural cell to endothelial cell ratio is approximately 1:1. In the intermediate part of the retina zebrafish had on average 4472.38 ± 719.57 endothelial cells per mm^2^ capillary area which translated to a mural cell to endothelial cell ratio of 1.36:1 (Figure 6).

Thus, zebrafish have a higher mural cell to endothelial cell ratio than mammals. This finding needs further investigation, as at this point it is unclear why zebrafish need a higher number of mural cells in the retina.

## 5 Discussion

In the present study, we showed that the different cell types of the mammalian retinal vasculature can be identified and quantified in the zebrafish retina with only slight alterations to established protocols (Dietrich and Hammes, 2012) using only Mayer’s hemalum nuclei stain. Endothelial cells and mural cells have distinct morphologies and locations in the zebrafish retinal trypsin digest preparation. In comparison to the recently published protocol (Lee, et al., 2022), we report a method which will enable future researchers to analyze the zebrafish retina regardless of the transgenic line that is used in their experiments and without the need for antibody staining.

Furthermore, we quantified endothelial and vascular mural cells in the zebrafish retina for the first time. One key finding of our study was that the number of mural cells was higher in the zebrafish retina than it is in the mammalian retina. The number of endothelial cells in the rodent retina is comparable to the number of endothelial cells in the zebrafish retina. Humans and non-human primates have on average less retinal endothelial cells than zebrafish (Hammes, 2018). Interestingly, while in mammals the relation of mural cells to endothelial cells in the retinal vasculature has been shown to be 1:1 (Pfister, et al., 2013), in zebrafish the relation is approximately 1.4 mural cells to 1 endothelial cell. Quantitatively, zebrafish have a much higher number of mural cells in the retina than mammals.

In mammals, the mural cell to endothelial cell coverage differs from organ to organ (Sims, 1986). Vessel stability is dependent on the interaction of endothelial cells with vascular mural cells. Mural cells most likely promote vessel stability through molecular interaction with endothelial cells (von Tell, et al., 2006)]. It has been suggested that vascular mural cells may contribute to the blood-brain barrier and that they could function as a sensor for the metabolic surroundings of the retina (Armulik, et al., 2005).

It is not yet clear why zebrafish have a need for an increased number of mural cells in the retina. While it has been observed that vascular mural cells in the zebrafish retina are in direct contact with endothelial cells (Alvarez, et al., 2007), it is unclear whether interaction between mural cells and endothelial cells in the zebrafish retina is comparable to the mammalian retina. Interestingly, while hyperglycemia leads to an angiogenic phenotype in the zebrafish retina (Ali, et al., 2020; Wiggenhauser, et al., 2020), the involvement of other retinal cell types as expected from mammalian models of DR could not be identified (Schmitner, et al., 2021). Therefore, further research is needed to investigate the interactions of mural cells and endothelial cells in the zebrafish retina.

To provide comparability between zebrafish and mammals for this study we decided to calculate the capillary area using the vessel diameter. This led to the confirmation that most of the vessels in the adult zebrafish retina qualify as arterioles (diameter >10 µm) rather than capillaries, as shown before (Alvarez, et al., 2007).

It has been described that vascular mural cells change their phenotype and marker expression dependent on which vessel type they are associated with (Hartmann, et al., 2015; Holm, et al., 2018). Typically, arterioles are covered by vascular smooth muscle cells (vSMC). Although not generally recognized as a factor in the pathogenesis of DR, vSMC and arterioles have been found to be affected by hyperglycemic conditions as well (vom Hagen, et al., 2005; Gardiner, et al., 2007).

Apart from vSMC loss, persistent dilation of retinal arterioles has been also observed in diabetes, which may lead to increased retinal blood flow and may represent arteriolar dysfunction (Gardiner, et al., 2007). Interestingly, a vasodilatory phenotype has been observed in the hyaloid vasculature of hyperglycemic *pdx1* ^*−/−*^ zebrafish at 5 days post fertilization. This phenotype could be rescued through antihyperglycemic and antiangiogenic pharmacological interventions, indicating that the mechanism may be observed and studied in zebrafish models of DR at an early stage (Wiggenhauser, et al., 2020).

Furthermore, in the *pdx1* ^*−/−*^ model of DR it was shown that mutants expressed less Transgelin1 than controls from the age of 3 mpf onwards (Ali, et al., 2020). Transgelin1 is an early marker for vascular mural cells (vMC) in general (Santoro, et al., 2009), further indicating that vSMC may be affected by hyperglycemic conditions as well.

Another striking feature of the adult zebrafish retina is the different vessel densities according to retinal area. The reasons for this observation remain unknown. In zebrafish larvae, photoreceptor distribution is unisotropic, which may cause different vascular densities, but in adult zebrafish retinae, photoreceptors are organized in a highly regular array (Franke, Maia Chagas et al., 2019). Thus, it seems unlikely, that a regular photoreceptor distribution in adult eyes causes different vascular densities. One could speculate that the high regenerative potential of the zebrafish retina demands higher oxygen supply and thus causes a regional increase in vessel density. However, this idea awaits further investigations.

Performing the retinal trypsin digest method on the zebrafish retina may help researchers analyze the role of arterioles and vSMC in the pathogenesis of diabetic retinopathy.

Another key difference that needs to be considered when applying the retinal trypsin digest protocol to the zebrafish retina is the anatomy. In mammals, the retinal vascular network is the result of coupled hyaloid regression and retinal angiogenesis leading to two vascular plexuses, the choroid and the intraretinal vasculature (Provis, 2001; Hammes, 2018). In zebrafish the initial hyaloid vasculature which encircles the lens does not regress but is remodeled to be attached to the retina (Alvarez, et al., 2007). There are no intraretinal plexus within the zebrafish retina and the vasculature remains on top of the ILM (Alvarez, et al., 2007). It is likely that there is no need for intraretinal plexuses because of the thinner diameter of the zebrafish retina. However, it is necessary to consider the anatomical differences, as removal of the ILM as done in the mammalian retina before the trypsin digest will lead to loss of the vasculature in zebrafish.

In conclusion, we have further characterized the zebrafish retinal vasculature using the retinal trypsin digest protocol and we provide a novel statistical quantification of the vasculature and its cell types. We found that the different vascular cell types can be identified through morphology and location and by applying the quantification protocol, we report novel vascular features within the physiological retina. In addition, quantification of the cell numbers showed that zebrafish have an increased number of vascular mural cells in comparison to the mammalian retina and an increased mural cell to endothelial cell ratio. We have recently successfully applied the protocol showing an increased vessel diameter and reduced mural cell coverage in adult *aldh2.1* ^*−/−*^ retinae proving the usefulness of the protocol (Wohlfart, et al., 2022). Thus, the protocol will help researchers in the field to analyze the zebrafish retina for signs of DR or other retinal diseases through the possibility of quantifying vascular cell numbers as well as vessel dilation.

## Data Availability

The original contributions presented in the study are included in the article/Supplementary material, further inquiries can be directed to the corresponding author.
